# Impact of sarcopenia and fat distribution on outcomes in penile cancer

**DOI:** 10.1038/s41598-024-73602-6

**Published:** 2024-10-25

**Authors:** Valerie Hartmann, Simon Udo Engelmann, Christoph Pickl, Maximilian Haas, Sebastian Kälble, Christopher Goßler, Christoph Eckl, Aybike Hofmann, Renate Pichler, Maximilian Burger, Roman Mayr

**Affiliations:** 1 Department of Urology, St. Josef Medical Center, University Medical Center Regensburg, Regensburg, Germany; 2 Department of Pediatric Urology, Clinic St. Hedwig, University Medical Center Regensburg, Regensburg, Germany; 3 Department of Urology, Comprehensive Cancer Center, Medical University of Innsbruck, Innsbruck, Austria

**Keywords:** Squamous carcinoma, Body composition, Skeletal muscle index, Psoas muscle index, Visceral obesity, Oncology, Urology

## Abstract

Sarcopenia, defined as age-associated loss of skeletal muscle function and muscle mass, is a negative prognostic marker for survival in several tumor entities. However, data evaluating the impact of sarcopenia and fat distribution on penile cancer are rarely described. We performed a retrospective study including 38 patients who were diagnosed with squamous cell carcinoma of the penis. By measuring skeletal muscle mass and fat distribution at axial abdominal computed tomography images at the third lumbar vertebra several body composition parameters including skeletal muscle index (SMI), psoas muscle index (PMI), visceral obesity and visceral-to-subcutaneous fat ratio were determined. Among 38 patients, 26% (*n* = 10) of the patients with penile cancer were identified as sarcopenic. SMI, age, lymph node metastases, distant metastases and penile cancer of the shaft were identified as significant risk factors for overall survival. PMI and distant metastases were significantly associated with cancer specific survival. None of the analysed adipose tissue parameters could be identified as risk factors for survival in this study. We showed that sarcopenia occurs in a relevant part of patients with penile cancer and is a significant risk factor for overall survival (*p* = 0.032) and cancer specific survival (*p =* 0.034) for patients with penile cancer. Regarding fat distribution further studies are needed to evaluate its impact on sarcopenia and survival.

## Introduction

Penile cancer is a rare disease with a worldwide incidence of 0.8 per 100.000 person-years but shows wide variation across the globe. Although incidence in Europe is low, it has been steadily increasing over the past few decades^1^. Increased age of the population, decreasing rates of circumcision in children and the increase in human papillomavirus prevalence are discussed as reasons for the increased risk of penile cancer^1,2^. Along with increasing incidence rates, the survival of patients with penile cancer is simultaneously decreasing^3^. However, due to the rarity of penile cancer there are only a few studies addressing this effect.

Sarcopenia, defined as age-associated loss of skeletal muscle function and muscle mass, is well recognized as a prognostic marker for survival in cancer patients^4^. For several tumor entities, including urological malignancies, sarcopenia has been investigated as a negative prognostic factor for postoperative complications and survival^5–9^. For penile cancer only two studies evaluating the impact of sarcopenia on survival and postoperative complications have been published^10,11^. The most common parameters to calculate sarcopenia are SMI and PMI^12–16^. In addition to sarcopenia, several other body composition parameters are being studied, that may have an impact on the prognosis of cancer patients^5,17,18^. They mostly relate to body fat distribution, such as visceral obesity (VO), visceral fat index (VFI), subcutaneous fat index (SFI) and visceral-to-subcutaneous fat ratio (VSR)^19–21^. Previous studies showed that fat distribution in favor of visceral fat is associated with several medical disorders and malignancies including prostate, breast, hepatocellular and colorectal cancer and affects survival^21,22^.

To the best of our knowledge, studies investigating the impact of muscle mass and body fat distribution parameters on the survival of penile cancer are lacking. Therefore, we conducted this study to examine associations of body composition parameters obtained from computed tomography (CT) images of patients with penile cancer.

## Patients and methods

### Patients

Ethical approval was granted by the institutional ethics committee of the university Regensburg (approval number: 21-2420-104) and the conducted research was performed in accordance with the relevant regulations and guidelines. No informed consent was obtained from the human participants, as the need for informed consent was waived by the ethics committee of the university Regensburg (Art. 27 (4) of the Bavarian Hospital Act).

We retrospectively reviewed patients who underwent surgical treatment due to penile cancer from 01.01.2010 to 31.12.2020 at our institution. In 68 patients squamous cell carcinoma of the penis was histologically verified. Imaging was performed by CT scan of the abdomen in 49 patients and by magnetic resonance imaging (MRI) in 3 patients. Staging wasn’t performed in 16 patients and in 11 patients staging was performed but was not available as digital images. Finally, 38 patients (56%) with available digital CT images and squamous cell carcinoma of the penis were included in the study.

Patient demographic data and comorbidities were collected from in-hospital medical records, including age, body mass index (BMI), American Society of Anesthesiologists physical status classification system (ASA), Charlson comorbidity index (CCI), alcohol abuse, insurance status, tumour localization, the presence of phimosis, smoking status, diabetes mellitus and renal function. Information about surgery, especially the type of resection, was also recorded. Moreover, we collected data about lymph node dissection. In this study collective modified and radical inguinal lymph node dissection and pelvic lymph node dissection were performed. In addition, we reviewed histopathological data including TNM classification.

Follow-up data was collected from our outpatient department by reviewing the medical records. The data collected included follow-up date, date of death, and cause of death. Overall survival (OS) was defined as time of diagnosis to death, irrespective of the cause of death. Cancer-specific survival (CSS) was defined as time from diagnosis to death, in case the event occurred due to the underlying penile cancer.

### CT image analysis and body composition measurements

Axial abdominal CT images at the level of the third lumbar vertebra (L3) were used to determine body composition parameters as previously described^5^. Measurements were performed using Osirix DICOM Viewer software (OsiriX MD version 13.0.0, Pixmeo, Geneva, Switzerland). The “Grow Region (2D/3D Segmentation)” tool was used to automatically select the required tissue in one axial image. If necessary, the selected area was corrected manually. The measurements were performed at two continuous axial CT images on which both vertebral spines were visible and the average was calculated.

**Fig. 1:**
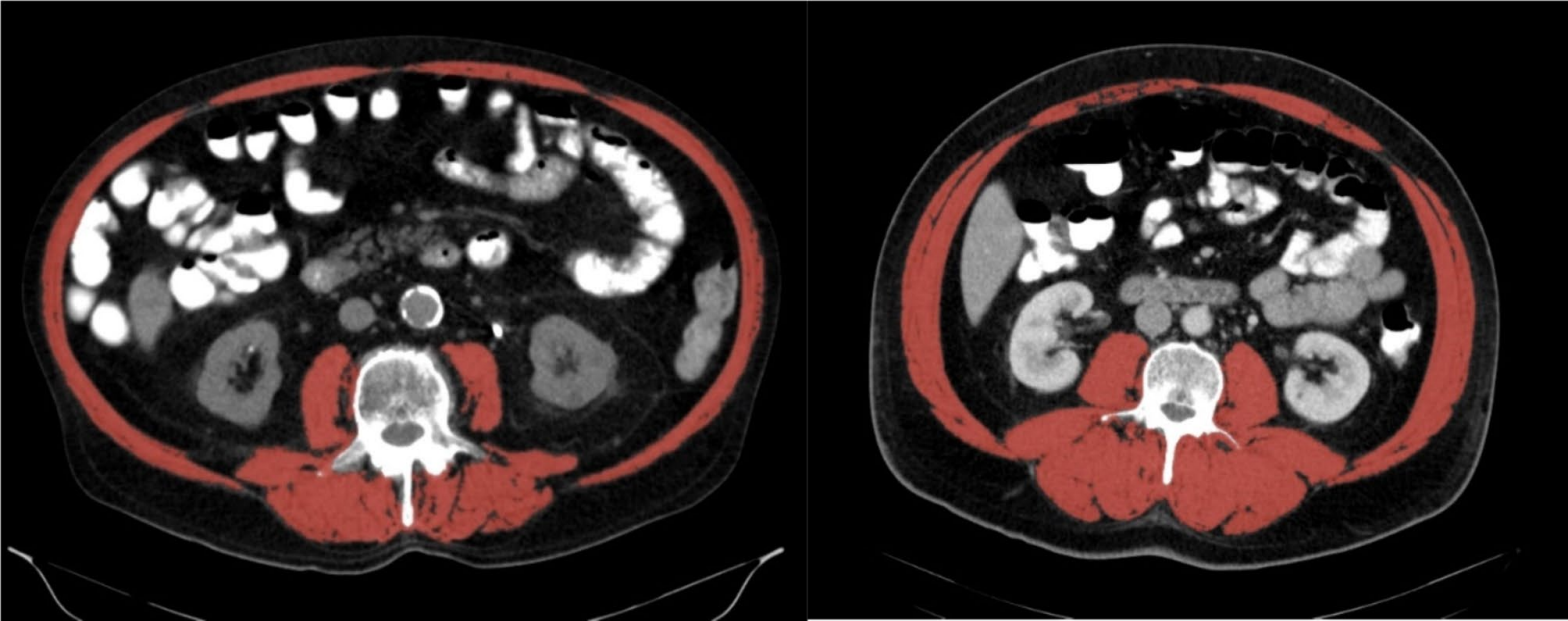
Illustration of axial CT images at the third lumbar vertebra with highlighting of the skeletal muscles by selecting the appropriate HU. Left illustration shows a sarcopenic patient (SMI 44,3 cm^2^/m^2^), right illustration shows a non-sarcopenic patient (SMI 81,4 cm^2^/m^2^).

The skeletal muscles in the L3 region include psoas, erector spinae, quadratus lumborum, transversus abdominis, external and internal obliques and rectus abdominis (Fig. 1). To differentiate the skeletal muscles from other tissue, a threshold range of Hounsfield units (HU) of -29 to + 150 was used^23^. The cross-sectional skeletal muscle surface (cm^2^) was normalized for height in meters squared (m^2^) to obtain the skeletal muscle index (SMI) (cm^2^/m^2^). Sarcopenia was defined as previously described by Martin et al. (males SMI ≤ 43 cm^2^/m^2^ if BMI < 25 and ≤ 53 cm^2^/m^2^ for all other BMI values)^12^.

Psoas muscle index (PMI) (cm^2^/m^2^) was computed by normalizing the cross-sectional psoas muscle surface (cm^2^) for height in meters squared (m^2^)^15^. Due to the absence of suitable cut-off-parameters for PMI the median was calculated and the cohort was divided into high and low PMI.

Measurements of subcutaneous and visceral fat tissue were also performed at the level of the third lumbar vertebra as described above. Fat tissue was separated from other tissues by a Hounsfield unit threshold range of − 150 to − 50. Visceral adipose tissue (cm^2^) and subcutaneous adipose tissue (cm^2^) were normalized for height in meters squared (m^2^), resulting in VFI (cm^2^/m^2^) and SFI (cm^2^/m^2^). VSR was calculated by dividing visceral fat area by subcutaneous fat area^23^. VO was defined as visceral fat area greater than 163.8 cm^2^^20^.

Since there are no standardized cut-off parameters for VFI, SFI and VSR the continuous values were used for further calculations. To be able to perform survival analysis for VSR, the cut-off for VSR was used as described by Engelmann et al. (males VSR > 1.421)^5^.

### Statistical analysis

Statistical analysis was performed using SPSS software version 28.0 (IBM, Armonk, NY, USA). Graphs were created using the software GraphPad Prism version 10.2.0 (GraphPad, San Diego, CA, USA) for Windows. Descriptive statistics are reported as median with interquartile range (IQR) or absolute numbers with percentages. Differences between the sarcopenic and non-sarcopenic group were analyzed using the chi-square test for dichotomous parameters and the Wilcoxon-Mann–Whitney U test for non-normally distributed data. Univariable Cox regression analyses were used to identify significant prognostic factors of OS and CSS. Kaplan-Meier curves were used to illustrate OS and CSS. P-values < 0.05 were considered statistically significant. All analyses were considered two-tailed.

## Results

### Descriptive data

The cohort in this study comprised 38 men with penile cancer with a median age of 64 years (IQR 56–72) at diagnosis. Eighteen (47%) patients had been diagnosed with phimosis. The surgical treatment for penile cancer included biopsy (2.6%), laser ablation (7.9%), circumcision (7.9%), partial penectomy (60.5%) and total penectomy (18.4%). Inguinal lymph node dissection (ILND) was performed in 26 (68.4%) patients. Regarding the site of penile cancer, 23 (60.5%) cases were located at the glans, 8 (21.1%) at the shaft, 2 (5.3%) at the foreskin and 5 (13.2%) cases were located multilocular. All cases of penile cancer histologically corresponded to squamous cell type and were classified as follows: 10.5% Carcinoma in situ, 36.8% T1, 26.3% T2 and 26.3% T3. Grading, based on the world health organization (WHO) classification, was described as follows: G1 8.6%, G2 51.4% and G3 40%. Lymph node metastases were detected in 17 (44.7%) cases, which were classified as follows: N1 15.8%, N2 18.4% and N3 10.5%. Distant metastases were detected in 3 (7.9%) cases. Ten patients underwent systemic chemotherapy (26.3%). The median BMI was 28 kg/m^2^ (IQR 24.6–31.6), which is classified as overweight. Further descriptive data is shown in Table 1.

**Table 1:** Patient characteristics of the entire cohort, sarcopenic patients and non-sarcopenic patients.

Characteristics	Entire Cohort	Sarcopenic	Non-sarcopenic	*p*-Value
	*n* = 38 (%)	*n* = 10 (26.3%)	*n* = 28 (73.7%)	
Median age at diagnosis (IQR)	64.5 (56.3–72.3)	76.5 (70.3–80.5)	59.5 (52.3–70)	**< 0.001**
Median SMI (IQR)	56.4 (49.2–65.6)	48 (42.9–50.2)	60.7 (55.3–67)	**< 0.001**
Median PMI (IQR)	7.4 (6.2–8.4)	6.9 (6.1–7.6)	7.7 (6.6-9)	0.127
Low PMI, *n* (%)	20 (52.6%)	7 (70%)	13 (46.4%)	0.200
Median VSR (IQR)	1.1 (0.7–1.6)	1.5 (1.2–2.1)	1 (0.6–1.4)	**0.022**
High VSR, *n* (%)	12 (31.6%)	6 (60%)	6 (21.4%)	**0.024**
Median VFI (IQR)	76 (42.6–97.4)	86.6 (66.6–99)	70.6 (39.6–96.3)	0.220
Median SFI (IQR)	59.4 (41.2–76.3)	60.6 (40.4–82.8)	55.4 (40.5–76.7)	0.791
High VO, *n* (%)	27 (71.1%)	9 (90%)	18 (64.3%)	0.124
pT-stage, *n* (%)				
pCis	4 (10.5%)	0 (0%)	4 (14.3%)	0.513
pT1	14 (36.8%)	3 (30%)	10 (35.7%)	
pT2	10 (26.3%)	3 (30%)	7 (24.9%)	
pT3	10 (26.3%)	4 (40%)	6 (21.4%)	
pN-stage, *n* (%)				
pN0	21 (55.3%)	4 (40%)	17 (60.7%)	0.593
pN1	6 (15.8%)	2 (20%)	4 (14.3%)	
pN2	7 (18.4%)	2 (20%)	5 (17.9%)	
pN3	4 (10.5%)	2 (20%)	2 (7.1%)	
cM-stage, *n* (%)	3 (7.9%)	0 (0%)	3 (10.7%)	0.281
Median BMI (IQR)	27.9 (24.6–31.6)	27.8 (25.2–30.3)	28.6 (24.5–31.7)	0.740
BMI, *n* (%)				
Underweight (< 18.5)	0 (0%)	0 (0%)	0 (0%)	0.181
Normal (18.5–24.9)	11 (28.9%)	2 (20%)	9 (32.1%)	
Overweight (25–29.9)	14 (36.8%)	6 (60%)	8 (28.6%)	
Obese (30–34.9)	13 (43.2%)	2 (20%)	11 (39.3%)	
Phimosis, *n* (%)	18 (47.3%)	6 (60%)	12 (42.8%)	0.351
ASA-Score, *n* (%)				
1	6 (15.8%)	2 (20%)	4 (14.3%)	0.277
2	19 (49.9%)	3 (30%)	16 (57.1%)	
3	13 (34.2%)	5 (50%)	8 (28.6%)	
Smoker, *n* (%)	12 (31.6%)	3 (30%)	9 (32.1%)	0.900
Cancer localization, *n* (%)				
Foreskin	2 (5.3%)	0 (0%)	2 (7.14%)	0.156
Glans	23 (60.5%)	4 (40%)	19 (67.9%)	
Shaft	8 (21.1%)	4 (40%)	4 (14.3%)	
Multilocular	5 (13.2%)	2 (20%)	3 (10.7%)	
Diabetes mellitus, *n* (%)	7 (18.4%)	2 (20%)	5 (17.9%)	0.881
Private insurance	11 (28.9%)	3 (30%)	8 (28.6%)	0.932
Alcohol abuse	4 (10.4%)	2 (20%)	2 (7.1%)	0.255
Chemotherapy, *n* (%)	10 (26.3%)	8 (80%)	2 (7.14%)	0.882
CCI				
0	4 (10.5%)	0 (0%)	4 (14.3%)	0.206
1–2	13 (34.2%)	1 (10%)	12 (42.8%)	0.060
3–4	14 (36.8%)	5 (50%)	9 (32.1%)	0.315
≥ 5	7 (18.4%)	4 (40%)	3 (10.7%)	**0.040**
LND				
None	12 (13.6%)	2 (20%)	10 (35.7%)	0.359
Modified	11 (28.9%)	2 (20%)	9 (32.1%)	0.467
Radical	5 (13.2%)	1 (10%)	4 (14.3%)	0.731
Pelvin	10 (26.3%)	5 (50%)	5 (17.9%)	**0.048**

*n* = count of patients (percentage); IQR, interquartile range; SMI, skeletal muscle index; PMI, psoas muscle index; VSR, visceral-to-subcutaneous fat ratio; VFI, visceral fat index; SFI, subcutaneous fat index; VO, visceral obesity; pT-stage, pathological Tumor stage; pN-stage, pathological nodal classification; cM-stage, clinical metastases classification; BMI, body mass index; ASA, American Society of Anesthesiologists. CCI, Charlson comorbidity index; LND, lymph node dissection; *p* < 0.05 is considered statistically significant.

### Impact of sarcopenia on demographic and tumor characteristics

The median SMI was 56.4 cm^2^/m^2^ (IQR 49.2–65.6 cm^2^/m^2^). Ten patients (26.3%) were classified as sarcopenic. With a median age of 76.5 years (IQR 70.3–80.5) vs. 59.5 years (IQR 52.3–70.0) (*p* < 0.001), those patients were significantly older. Furthermore, significantly more sarcopenic patients were classified with a high VSR (60% vs. 21%, *p* = 0.024). No significant differences were found between both groups regarding BMI, ASA, VFI, SFI, VO, tumour localization, TNM classification, lymph node metastases, distant metastases, presence of phimosis, smoking status, diabetes mellitus and renal function (Table 1).

There was no difference in terms of t-stage between patients who received staging and those who did not and therefore had to be excluded from the study (*p =* 0.731).

### Survival analysis

The median observation time was 47 months (IQR 14.3–73.4). At the time of analysis 27 (71%) patients were alive and 11 (29%) patients had died. In univariate cox regression analysis age, sarcopenia (SMI), lymph node metastases, distant metastases and penile cancer of the shaft were significantly associated with decreased OS (Table 2). The continuous parameters SMI (cm^2^/m^2^) and PMI (cm^2^/m^2^) showed a tendency towards statistical significance (*p* = 0.065 and *p* = 0.067, respectively) concerning OS.

**Table 2:** Univariate Cox Regression analysis for overall survival in patients with penile cancer.

Characteristics	HR	95%-CI	*p*-Value
Age at diagnosis	1.09	1.02–1.17	**0.011**
Presence of sarcopenia (SMI)	3.54	1.02–12.3	**0.046**
Presence of sarcopenia (PMI)	2.3	0.59–8.93	0.227
SMI (continous)	0.93	0.86-1.00	0.065
PMI (continous)	0.67	0.43–1.03	0.067
VSR (continous)	1.83	0.51–6.48	0.350
High VSR	2.43	0.70–8.39	0.161
VFI	0.99	0.98–1.01	0.386
SFI	0.98	0.95–1.01	0.109
VO	0.95	0.25–3.67	0.938
pT-stage			
pTis	0.41	0-18.12	0.455
pT1	1.05	0.30–3.74	0.935
pT2	0.67	0.14–3.14	0.608
pT3	2.36	0.66–8.37	0.186
pN-stage	6.55	1.38-31	**0.018**
cM-stage	7.41	1.83–29.92	**0.005**
BMI	1.11	0.97–1.26	0.132
BMI categorized			
Normal	0.58	0.12–2.73	0.490
Overweight	0.65	0.17–2.52	0.533
Obese	2.23	0.67–8.03	0.184
Phimosis	1.84	0.52–6.52	0.347
ASA-Score			
1	1.20	0.26–5.65	0.818
2	0.41	0.11–1.59	0.197
3	2.16	0.63–7.48	0.224
Smoker	0.80	0.21–3.10	0.749
Localization			
Foreskin	0.45	0.00-4173.15	0.595
Glans	0.53	0.15–1.84	0.320
Shaft	3.63	1.02-13.00	**0.047**
Multilocular	0.75	0.10–5.90	0.781
Diabetes mellitus	1.13	0.24–5.33	0.877
Private insurance	0.57	0.11–2.67	0.472
Alcohol abuse	0.79	0.10–6.28	0.827
Median Creatinine (mg/dl) (IQR)	0.80	0.21–3.10	0.749
Chemotherapy	1.89	0.48–7.34	0.380
CCI			
0	0.89	0.11–7.03	0.911
1–2	0.46	0.1–2.17	0.328
3–4	2.94	0.82–10.42	0.096
≥ 5	0.45	0.06–3.53	0.445
LND			
None	0.5	0.11–2.37	0.385
Modified	0.6	0.13–2.83	0.518
Radical	0.81	0.1–6.36	0.837
Pelvin	2.96	0.86–10.26	0.086

HR, hazard ratio; CI, confidence interval; SMI, skeletal muscle index; PMI, psoas muscle index; VSR, visceral-to-subcutaneous fat ratio; VFI, visceral fat index; SFI, subcutaneous fat index; VO, visceral obesity; pT-stage, pathological Tumor stage; pN-stage, pathological nodal classification; cM-stage, clinical metastases classification; BMI, body mass index; ASA, American Society of Anesthesiologists. CCI, Charlson comorbidity index; LND, lymph node dissection; *p* < 0.05 is considered statistically significant.

Moreover, continuous PMI (cm^2^/m^2^) and distant metastases were significantly associated with CSS. For low PMI, age and penile cancer of the shaft univariate analysis revealed a trend towards statistical significance (*p* = 0.069 and *p* = 0.096, respectively) concerning CSS (Table 3).

**Table 3:** Univariate Cox Regression analysis for cancer specific survival in patients with penile cancer.

Characteristics	HR	95%-CI	*p*-Value
Age at diagnosis	1.07	0.997–1.15	0.061
Presence of sarcopenia (SMI)	2.21	0.52–9.28	0.278
Presence of sarcopenia (PMI)	7	0.86–57.09	0.069
SMI (continous)	0.95	0.88–1.03	0.220
PMI (continous)	0.57	0.34–0.96	**0.037**
VSR (continous)	1.23	0.31–4.91	0.773
High VSR	1.48	0.35–6.19	0.593
VFI	0.99	0.97–1.01	0.443
SFI	0.98	0.96-1.0	0.249
VO	0.67	0.16–2.82	0.589
pT-stage			
pTis	0.04	0.00-453.3	0.500
pT1	0.95	0.23–3.98	0.945
pT2	0.88	0.18–4.36	0.875
pT3	2.16	0.51 − 0.06	0.293
pN-stage	125.39	0.29-53405.57	0.118
cM-stage	11.47	2.53–52.05	**0.002**
BMI	1.1	0.94–1.27	0.228
BMI cat.			
Normal	0.76	0.15–3.79	0.742
Overweight	0.50	0.10–2.49	0.399
Obese	2.37	0.59–9.47	0.224
Phimosis	2.06	0.49–8.46	0.324
ASA-Score			
1	1.59	0.32–7.86	0.573
2	0.32	0.06–1.57	0.148
3	2.20	0.55–8.83	0.266
Smoker	1.11	0.26–4.63	0.891
Localization			
Foreskin	0.05	0.00-13975.82	0.631
Glans	0.53	0.13–2.11	0.365
Shaft	3.4	0.80-14.37	0.096
Multilocular	0.95	0.12–7.72	0.961
DM	1.51	0.30–7.47	0.617
Private insurance	0.742	0.15–3.68	0.715
Alcohol abuse	0.99	0.12–8.11	0.996
Creatinine	1.106	0.26–4.63	0.891
Chemotherapy	2.70	0.64–11.35	0.201
CCI			
0	1.14	0.14–9.32	0.900
1–2	0.26	0.03–2.13	0.211
3–4	3.29	0.79–13.78	0.103
≥ 5	0.57	0.07–4.65	0.600
LND			
None	0.66	0.13–3.28	0.613
Modified	0.34	0.04–2.78	0.315
Radical	0.04	0.00-453.31	0.501
Pelvin	5.02	1.2-21.07	**0.027**

HR, hazard ratio; CI, confidence interval; SMI, skeletal muscle index; PMI, psoas muscle index; VSR, visceral-to-subcutaneous fat ratio; VFI, visceral fat index; SFI, subcutaneous fat index; VO, visceral obesity; pT-stage, pathological Tumor stage; pN-stage, pathological nodal classification; cM-stage, clinical metastases classification; BMI, body mass index; ASA, American Society of Anesthesiologists. CCI, Charlson comorbidity index; LND, lymph node dissection; *p* < 0.05 is considered statistically significant.

**Fig. 2:**
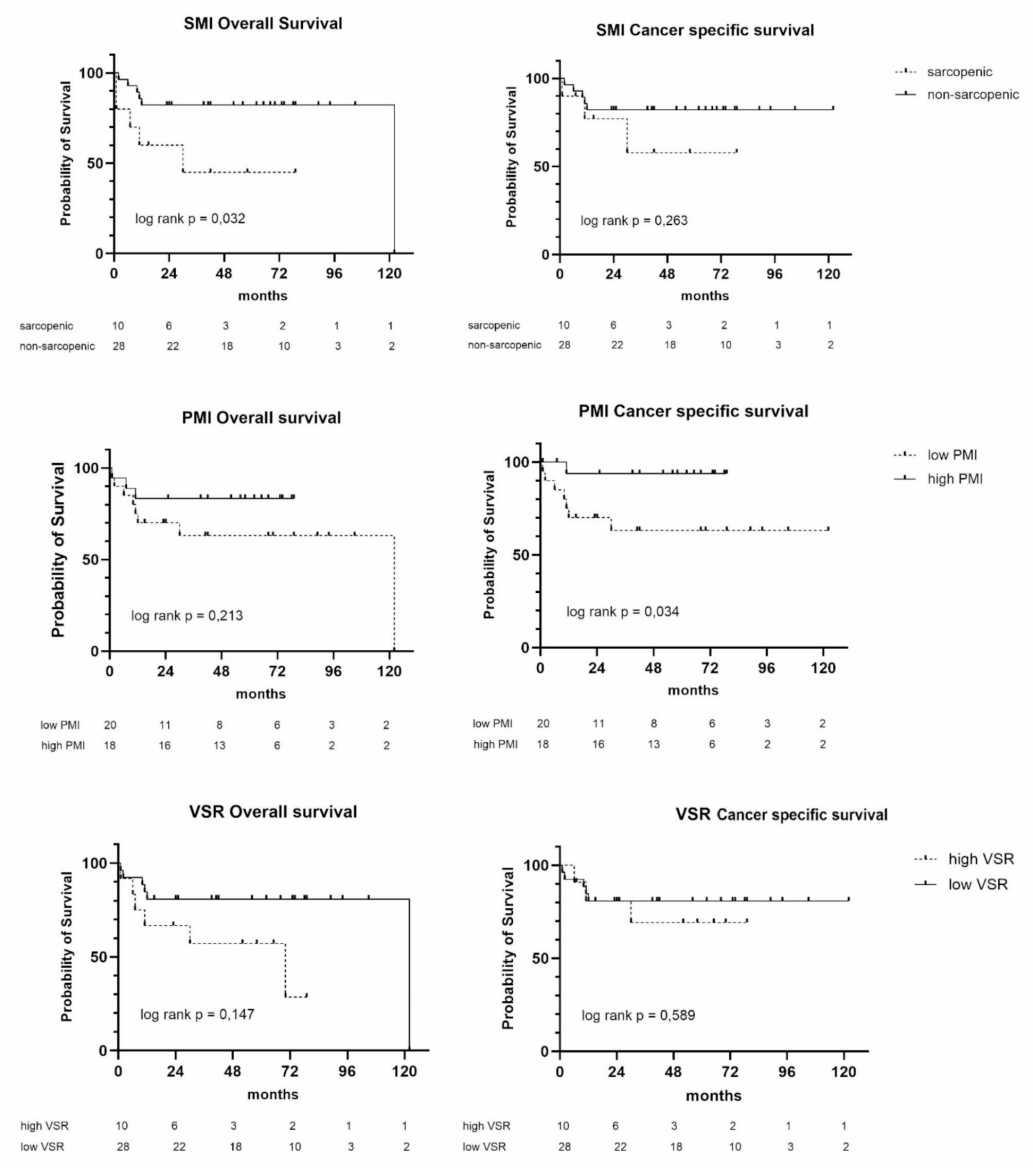
Kaplan–Meier survival curves for overall survival and cancer specific survival stratified by different body composition parameters (SMI, PMI, VSR). SMI, skeletal muscle index; PMI, psoas muscle index; VSR, visceral-to-subcutaneous fat ratio.

Kaplan-Meier curves for patients stratified by sarcopenia (SMI) showed a significantly lower OS for sarcopenic patients (41 vs. 101 months, *p* = 0.032). Kaplan-Meier curves for patients stratified by PMI showed a significantly lower CSS for patients with low PMI (74 and 81 months, respectively (*p* = 0.034). Further survival curves for SMI, PMI and VSR are illustrated in Fig. 2.

None of the analysed adipose tissue parameters (VFI, SFI, VSR, and VO) could be identified as risk factor for OS or CSS in this study cohort.

## Discussion

Sarcopenia was described in several studies as predictor for poorer survival, especially in patients with malignancies^7,24,25^. To date, only two studies have been published that investigated the prevalence and influence of sarcopenia in patients with penile cancer^10,11^. Therefore, we conducted this study and analyzed low skeletal muscle mass in patients with penile cancer retrospectively. Our results show a prevalence of sarcopenia of 26% among patients with penile cancer. Takkamoto et al. investigated sarcopenia in patients with penile carcinoma using the PMI and found that the prevalence of sarcopenia was comparable at 32%^11^. Sharma et al. even showed a prevalence of 51% for patients with penile carcinoma, who underwent lymph node dissection^10^. However, it should be noted that each study used different diagnostic criteria for sarcopenia. The Martin criteria, which we used in our study, were determined from 1473 cancer patients and represent a fairly accurate classification for sarcopenia^12^. Sharma et al. used cut-off values validated by the European working group on sarcopenia in older people in 2010^26^. Takkamoto et al. instead determined their own threshold values for PMI using receiver operating characteristic analysis (ROC)^11^. As there are several options to define and measure sarcopenia, it is quite difficult to compare results of different studies. Although the methods in these studies differ, their results are in line with our findings and confirm, that sarcopenia is found in a relevant proportion of patients with penile cancer and should be considered in treatment alongside targeted tumor therapy.

Furthermore, our data show that sarcopenia is a negative predictive factor for survival of patients with penile cancer. Univariate Cox regression analysis showed a significantly reduced OS for sarcopenia, calculated by SMI, and a significantly reduced CSS for sarcopenia, calculated by PMI. This is in line with several other studies that investigated the influence of sarcopenia on the survival of patients with solid tumor diseases^5–7,15,15,22,24^.

Beside sarcopenia, we showed, that also advanced age and distant metastases are significant risk factors for OS and CSS, respectively.

Due to the small number of patients, we were unable to perform a multivariate analysis that could identify independent risk factors. Nevertheless, we think that every published data contributes significantly to the existing state of knowledge, particularly in the context of rare diseases like penile cancer. For more reliable results we suggest performing further investigations within multicenter studies to create bigger patient cohorts as penile cancer is a relatively rare disease.

To the best of our knowledge, no comparable study has yet investigated the influence of fat distribution on the outcome of penile cancer. Especially visceral obesity is associated with medical disorders such as cardiovascular disease and several malignancies^21^. Moreover, sarcopenic obesity, defined by the co-existence of obesity and sarcopenia, was mentioned as risk factor for frailty, comorbidities and mortality^27^. Our data show that sarcopenic patients have a significantly higher VSR than non-sarcopenic patients. 60% of patients with sarcopenia were classified as high VSR, however only 21% of non-sarcopenic patients were classified as high VSR. These results show that sarcopenia should not only be treated as a single symptom, but as a complex of physical changes in elderly people. Although our study did not show any significant influence for OS and CSS regarding body fat distribution parameters, the graphical representation of the Kaplan Meier curves for VSR suggests a difference in survival between patients with high and low VSR.

In literature, there are conflicting results in terms of fat distribution and survival regarding other cancer entities. High visceral fat seems to affect survival among patients with colorectal, pancreatic and prostate cancer negatively^28,29^. Performing a systematic review, Lopez et al. found that high levels of subcutaneous fat and low levels of VAT/SAT were associated with a longer survival, in patients at advanced stages of prostate cancer^29^. On the other hand, high visceral fat was mentioned as protective factor for survival in patients with renal cell carcinoma and urothelial cancer of the upper urinary tract^6,29,30^. Lee HW et al. determined fat distribution of 2178 patients with renal cell carcinoma and showed that high visceral fat was associated with longer cancer-specific survival (*p* = 0.01) and overall survival (*p* = 0.03). Further research is required to assess the role of visceral and sarcopenic obesity in patients with penile cancer.

Beside the relatively small size of patients in this study, another limitation of our study is that due to the presented method only patients with available CT could be included in the study. Although we didn’t find any significant differences regarding t-stage of patients with and without staging, this diagnostic technique could influence the results by selecting patients in advance in retrospective studies. A conceivable method to assess skeletal muscle mass would be to determine psoas muscle mass using ultrasound. This portable, cheap and non-invasive method could be used especially for prospective studies to monitor skeletal muscle mass and sarcopenia, respectively, without any radiation exposure^31^.

Due to the retrospective design several diagnostic parameters relating to sarcopenia, such as muscle force measurement by hand-grip-strength and functional tests like chair-stand-test, as well as information about dietary habits, nutritional status and physical activity could not be recorded. There are several studies, that show that exercise and nutritional support program has the potential to reduce sarcopenia and improve outcome in elderly sarcopenic patients with cancer disease^32^. We believe that patients with penile cancer should also be specifically screened for secondary diseases such as sarcopenia. Also with regard to the shrinking budget of the healthcare system, this personalized approach not only improves patient outcomes but also optimizes the use of limited funds, ensuring that support is provided to those who will benefit most.

As we showed, sarcopenia is present in a relevant part of patients with penile cancer. Therefore, we suggest prospective studies to collect named data and develop nutritional- and exercise programs to improve live quality and survival alongside targeted tumor therapy.

## Conclusions

In conclusion, sarcopenia occurs in a relevant part of patients with penile cancer and is significantly associated with a reduced OS and CSS. Regarding fat distribution parameters we showed that VSR is associated with sarcopenia. Thus, beside low muscle mass also fat distribution, particularly high visceral fat, should be taken into focus for further studies. Additional prospective research is needed to evaluate whether early intervention of muscle mass maintaining and fat reduction, including exercise and specific nutritional optimization, may achieve better cancer management and thus, better outcome results.

## Data Availability

The datasets used and analyzed during the current study are available from the corresponding author on reasonable request.
